# Tracing the origin of the NS1 A188V substitution responsible for recent enhancement of Zika virus Asian genotype infectivity

**DOI:** 10.1590/0074-02760170299

**Published:** 2017-11

**Authors:** Edson Delatorre, Daiana Mir, Gonzalo Bello

**Affiliations:** Fundação Oswaldo Cruz-Fiocruz, Instituto Oswaldo Cruz, Laboratório de AIDS e Imunologia Molecular, Rio de Janeiro, RJ, Brasil

**Keywords:** Zika Virus, NS1, infectivity, evolution

## Abstract

A recent study showed that infectivity of Zika virus (ZIKV) Asian genotype was enhanced by an alanine-to-valine amino acid substitution at residue 188 of the NS1 protein, but the precise time and location of origin of this mutation were not formally estimated. Here, we applied a Bayesian coalescent-based framework to estimate the age and location of the ancestral viral strain carrying the A188V substitution. Our results support that the ancestral ZIKV strain carrying the A188V substitution arose in Southeastern Asia at the early 2000s and circulated in that region for some time (5-10 years) before being disseminated to Southern Pacific islands and the Americas.

Zika virus (ZIKV) is a mosquito-borne pathogen member of the family *Flaviviridae*, genus Flavivirus, that was first isolated from a sentinel monkey in Uganda in 1947 (Dick et al. 1952). Until recently, ZIKV was most likely maintained in a sylvatic cycle involving vectors of the genus *Aedes* and non-human African primates (Hayes 2009). Since 2007, however, large epidemics of ZIKV were described in human populations from the Pacific islands and more recently in the Americas (Gatherer & Kohl 2016).

Recent studies support that pandemic expansion of ZIKV Asian lineage was associated with viral adaptations in both mosquitoes and humans (Freire et al. 2015, Liu et al. 2017), offering a potential explanation for the successful spread of the virus along urban chains of transmission. Freire et al. (2015) described adaptation of the ZIKV Asian lineage NS1 codon usage to human housekeeping genes, which could facilitate viral replication in humans. More recently, Liu et al. (2017) demonstrate that ZIKV Asian lineage infectivity in *Aedes aegypti* was enhanced by an alanine(A)-to-valine(V) amino acid substitution at residue 188 of the NS1 protein, resulting in increased NS1 antigenemia in infected hosts that in turn promotes ZIKV infectivity and prevalence in mosquitoes.

The authors suggest that the Asian lineage of ZIKV acquired enhanced infectivity when it spread from the Southeastern Asia to the Southern Pacific around 2013, because residue 188 of the NS1 protein was alanine in ZIKV isolates from the Asian clade collected before 2012, but was mutated to valine in all isolates collected after 2013. This hypothesis, however, was not formally tested using a model-based statistical framework. Here, we performed a Bayesian evolutionary and phylogeographic analysis to reconstruct the spatiotemporal dissemination dynamics of the ZIKV Asian genotype and to properly estimate the age and location of the ancestral viral strain carrying the A188V substitution.

All near-complete ZIKV genome sequences from Asian, Southern Pacific and American countries with a known date of isolation were retrieved from GenBank on August 7th, 2017. This resulted in a final data set of 461 ZIKV Asian genotype genomes spanning a 50-year period, after excluding those sequences of imported cases with no information about country of infection. Complete coding sequences (CDS) were manually aligned and subjected to maximum likelihood (ML) phylogenetic reconstruction with PhyML v3.0 (Guindon et al. 2010), under the GTR+Γ4 nucleotide substitution model selected by jModelTest v1.6 (Posada 2008). The temporal signal of the dataset was verified using Tempest (Rambaut et al. 2016). The spatiotemporal viral diffusion pattern and the ancestral CDS at key internal nodes of the phylogeny were reconstructed using the Markov chain Monte Carlo (MCMC) algorithms implemented in BEAST v1.8 package (Drummond & Rambaut 2007). The temporal scale was estimated using a relaxed uncorrelated lognormal molecular clock model (Drummond et al. 2006), the GTR+Γ4 nucleotide substitution model and a Bayesian Skyline coalescent model (Drummond et al. 2005). Migration events throughout the phylogeny were reconstructed using both reversible (symmetric) and nonreversible (asymmetric) discrete phylogeographic models (Lemey et al. 2009). MCMC were run sufficiently long (20-100 million MCMC steps) to ensure stationary and convergence of all parameters (Effective Sample Size > 200), through inspection with Tracer v1.6 (http://tree.bio.ed.ac.uk/software/tracer/) after discarding the 10% burn-in. The maximum clade credibility (MCC) trees were generated and visualised with TreeAnnotator v1.8 and FigTree v1.4 (http://tree.bio.ed.ac.uk/software/figtree/), respectively. Consensus CDS at key ancestral nodes were computed using the R package SeqinR (http://seqinr.r-forge.r-project.org/).

ML phylogenetic analysis of 461 ZIKV Asian near-complete CDS revealed two highly supported (a*LRT* > 0.85) monophyletic clusters comprising all sequences from the Americas (n = 309) and from Singapore (n = 117) (Supplementary data, Fig. 1). Almost all ZIKV genomes from the Americas (except one sequence from Honduras) and all sequences from Singapore displayed the NS1 A188V substitution (Supplementary data, Fig. 1), thus supporting that this mutation arose before ZIKV dissemination to those locations. In order to reduce computation time, only representative subsets of sequences retaining most viral diversity information from each cluster (Americas and Singapore) were selected for further Bayesian analysis. To generate non-redundant ZIKV subsets from the Americas and Singapore, sequences from each location were grouped by similarity (≥ 99.9%) with the CD-HIT program (Li & Godzik 2006) using an online web server (Huang et al. 2010) and only one sequence per cluster was selected. Furthermore, sequences containing undetermined bases and American sequences sampled after 2015 were also removed. With this subsampling procedure, counts were reduced to a total of 65 ZIKV sequences from Southeastern Asia (n = 20, 1966-2016), Pacific (n = 23, 2007-2016) and the Americas (n = 22, 2014-2015).

Analysis of this balanced ZIKV subset reveals a very strong correlation (R^2^ = 0.99) between genetic divergence and sampling time within the ZIKV Asian lineage (Figure A), thus supporting the use of this subset for molecular clock calibration. The mean ZIKV evolutionary rate here estimated was roughly similar to those previously described (Faria et al. 2016, 2017, Pettersson et al. 2016, Metsky et al. 2017); but estimated ages of ZIKV Asian lineage ancestral nodes were slightly older (Supplementary data, Table). Phylogeographic analyses using both asymmetric (Figure B) and symmetric (Supplementary data, Fig. 2) diffusion models placed the most recent common ancestor of ZIKV Asian genotype epidemic strains in Southeastern Asia (posterior state probability, PSP = 1) at around 1999 [95% Bayesian credible interval (BCI): 1995-2003]. Reconstruction of ancestral ZIKV sequences at internal nodes traced the emergence of the NS1 A188V substitution in Southeastern Asia (PSP = 1) at some time between N2 [2003 (BCI: 2001-2005)] and N3 [2007 (BCI: 2005-2008)] (Figure B). A ZIKV Asian strain carrying the NS1 A188V substitution was later disseminated from Southeastern Asia (PSP = 1) to Southern Pacific islands at 2012 (BCI: 2012-2013) and from Southern Pacific (PSP = 1) into the Americas at 2013 (BCI: 2013-2013) (Supplementary data, Table).

**Figure:**
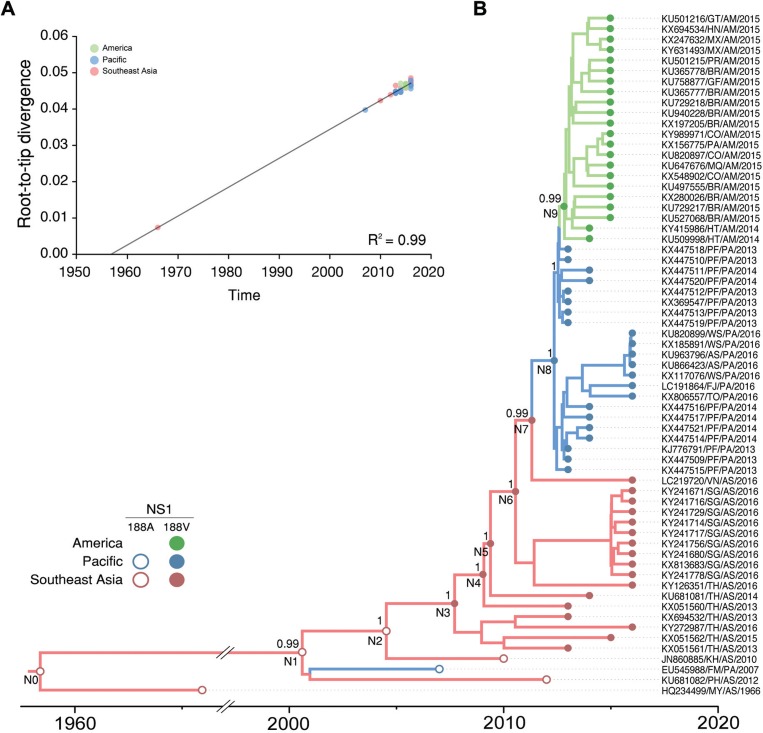
Emergence of the A188V substitution at NS1 protein during Zika virus (ZIKV) Asian genotype evolution. (A) Correlation between the sampling date of each ZIKV sequence (n = 65) and the genetic distance of that sequence from the root of a maximum likelihood (ML) phylogenetic tree. Colours indicate the geographic region of sampling. (B) Bayesian time-scale maximum clade credibility (MCC) phylogenetic tree estimated from ZIKV Asian genotype genomic sequences (n = 65). Branches are coloured according to the most probable location state (geographic region) of their descendent nodes as indicated at the legend on the lower left. Reconstructed ancestral key nodes and terminal nodes are highlighted with circles colored according to the location (colour) and the amino acid at NS1 188 residue (fill or empty), as indicated at the legend on the lower left. Numbers at key selected nodes represent the posterior probability supports of the clades. All horizontal branch lengths are drawn to a scale of years. Taxon labels include information of GenBank accession number, country of origin, region of origin (AM = Americas; AS = Asia; PA = Pacific) and year of isolation. Countries represented are: American Samoa (AS), Brazil (BR), Cambodia (KH), Colombia (CO), Federated States of Micronesia (FM), Fiji (FJ), French Guiana (GF), French Polynesia (PF), Guatemala (GT), Haiti (HT), Honduras (HN), Malaysia (MY), Martinique (MQ), Mexico (MX), Panama (PA), Philippines (PH), Puerto Rico (PR), Samoa (WS), Singapore (SG), Suriname (SR), Thailand (TH), Tonga (TO), and Vietnam (VN).

In summary, we showed that the NS1 A188V substitution associated with enhanced infectivity of ZIKV Asian lineage in *Ae. aegypti* mosquitoes probably arose during virus dissemination among urban chains of transmission in the Southeastern Asian region, between the early and the middle 2000s. Thus, ZIKV Asian genotype strains carrying the NS1 A188V mutation appear to have spread in the Southeastern Asian region for some time (5-10 years) before being disseminated to Southern Pacific islands and the Americas. The absence of the reversal NS1 V188A mutation at internal nodes in the ZIKV Asian genotype phylogeny and its extremely low frequency at terminal tips sampled after 2010, clearly supports some selective advantage for the fixation of the valine amino acid at residue 188 in NS1.
