# Supplementary material for: Tracing the origin of the NS1 A188V substitution responsible for recent enhancement of Zika virus Asian genotype infectivity
Source: Mem Inst Oswaldo Cruz. 2017 Nov;112(11):793–5. doi: 10.1590/0074-02760170299 (PMC5661894; doi:10.1590/0074-02760170299)
Supplement: Supplementary file 1 [file 0074-0276-mioc-0074-02760170299-suppl01.pdf]

TABLE

Posterior estimates of evolutionary parameters of Zika virus (ZIKV) Asian genotype

| | | | TMRCA[b] (95% BCI) | | |
|---|---|---|---|---|---|
| Study | Phylogeographic model | Evolutionary rate[a] (95% BCI) | Epidemic Asian clade | Southern Pacific clade | American clade |
| Faria et al. (2016) | - | $1.1\times10^{-3}$ ($8.5\times10^{-4}$ - $1.2\times10^{-3}$) | - | - | 2014.0 (2013.6-2014.3) |
| Pettersson et al. (2016) | - | $1.2\times10^{-3}$ ($1.1\times10^{-3}$ - $1.3\times10^{-3}$) | 2001.8 (2000.7-2002.8) | 2013.2 (2012.8-2013.4) | 2013.9 (2013.8-2014.2) |
| Faria et al. (2017) | - | $1.1\times10^{-3}$ ($9.7\times10^{-4}$ - $1.3\times10^{-3}$) | - | - | 2014.0 (2013.8-2014.3) |
| Metsky et al. (2017) | - | $1.2\times10^{-3}$ ($9.8\times10^{-4}$ - $1.3\times10^{-3}$) | - | - | 2014.0 (2013.7-2014.6) |
| This study | Reversible | $9.2\times10^{-4}$ ($7.1\times10^{-4}$ - $1.2\times10^{-3}$) | 1999.2 (1994.4-2002.8) | 2012.4 (2011.9-2012.7) | 2013.1 (2012.1-2013.4) |
| This study | Nonreversible | $9.2\times10^{-4}$ ($6.9\times10^{-4}$ - $1.2\times10^{-3}$) | 1999.2 (1994.6-2002.8) | 2012.4 (2011.9-2012.7) | 2013.1 (2012.7-2013.4) |

a: mean evolutionary rate (substitutions/site/year); b: median time of the most recent common ancestor (year); BCI: Bayesian credible interval.

**A**

| Location | Country | Maximum likelihood | | | Bayesian |
|---|---|---|---|---|---|
| | | *n* | NS1 188 | | *n* |
| | | | W/o"N"(%)* | Val (%)** | |
| America | BR | 100 | 91 (91.0) | 91 (100) | 10 |
| | CO | 14 | 12 (85.7) | 12 (100) | 3 |
| | CU | 2 | 2 (100) | 2 (100) | - |
| | DO | 36 | 36 (100) | 36 (100) | - |
| | EC | 2 | 2 (100) | 2 (100) | - |
| | GF | 1 | 1 (100) | 1 (100) | 1 |
| | GP | 1 | 1 (100) | 1 (100) | - |
| | GT | 3 | 3 (100) | 3 (100) | 1 |
| | HN | 23 | 20 (87.0) | 19 (95) | 1 |
| | HT | 10 | 10 (100) | 10 (100) | 1 |
| | JM | 4 | 4 (100) | 4 (100) | - |
| | MQ | 2 | 2 (100) | 2 (100) | 1 |
| | MX | 19 | 19 (100) | 19 (100) | 2 |
| | NI | 14 | 14 (100) | 14 (100) | - |
| | PA | 4 | 4 (100) | 4 (100) | 1 |
| | PE | 2 | 2 (100) | 2 (100) | - |
| | PR | 7 | 7 (100) | 7 (100) | 1 |
| | SR | 4 | 4 (100) | 4 (100) | - |
| | US | 57 | 54 (94.7) | 54 (100) | - |
| | VE | 4 | 4 (100) | 4 (100) | - |
| | Total | 309 | 292 (94.5) | 291 (99.7) | 22 |
| Asia | SG | 118 | 118 (100) | 118 (100) | 9 |

* Sequences without "N" in amino acid residue 188 of the NS1 protein
** Valine frequency calculated with sequences without "N"

**B**

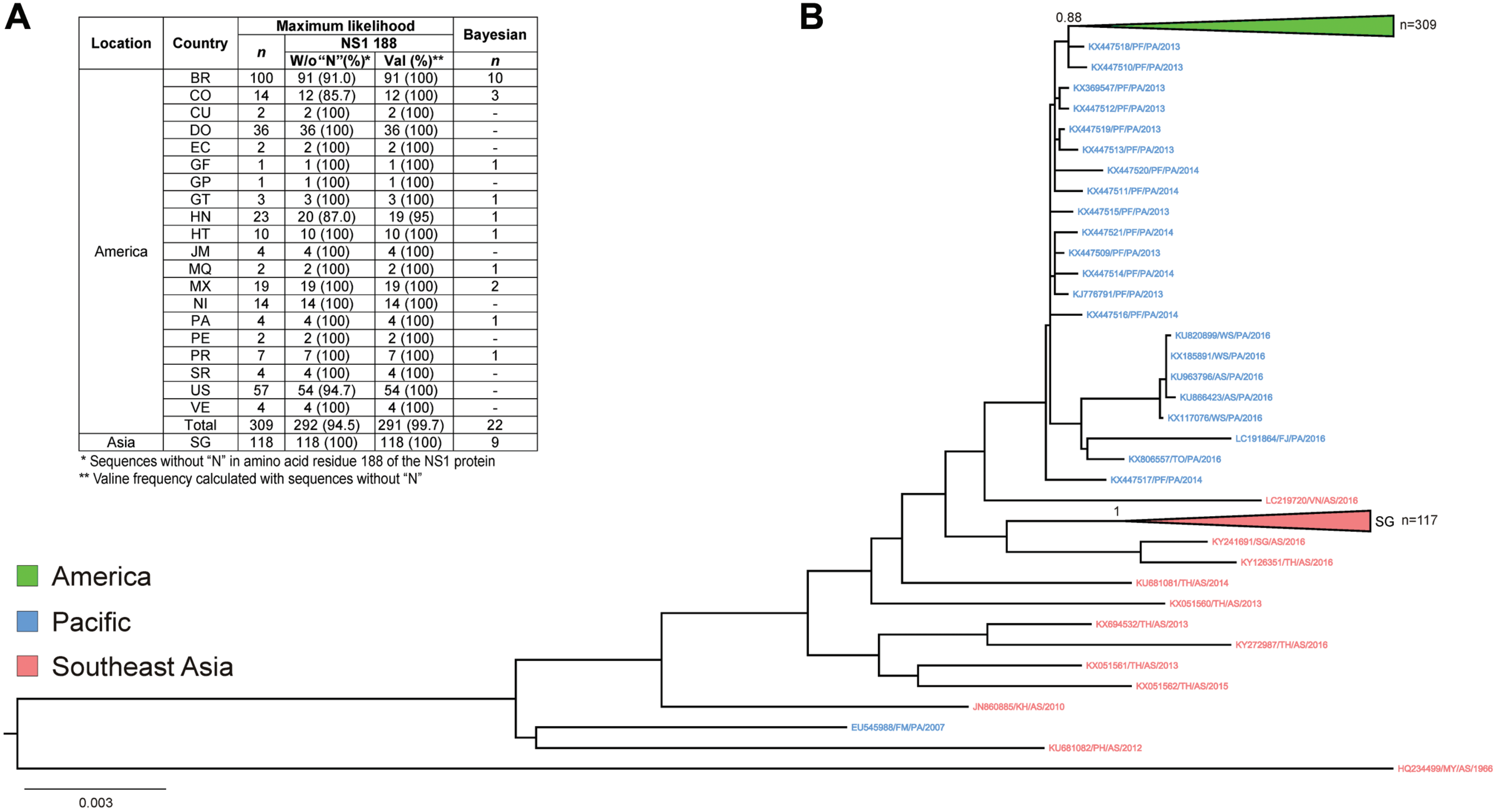


Fig. 1: Zika virus (ZIKV) Asian genotype sequence data. (A) Number of ZIKV sequences retrieved from GenBank belonging to the Americas and Singapore monophyletic clusters used for maximum likelihood (ML) and Bayesian inferences. (B) ML phylogenetic analysis of 461 ZIKV Asian near-complete coding sequences (CDS). Monophyletic clusters comprising all sequences of the Americas and Singapore have been collapsed. Tip labels and collapsed clusters are coloured according to the geographic origin of each sequence as indicated at the legend (lower left). The tree is midpoint rooted, and numbers denote a*LTR* support values of each collapsed cluster. Branch lengths are drawn to scale with the bar in the bottom indicating nucleotide substitutions per site. Country abbreviations represent: American Samoa (AS), Brazil (BR), Cambodia (KH), Colombia (CO), Cuba (CU), Dominican Republic (DO), Ecuador (EC), Federated States of Micronesia (FM), Fiji (FJ), French Guiana (GF), French Polynesia (PF), Guadeloupe (GP), Guatemala (GT), Honduras (HN), Haiti (HT), Jamaica (JM), Martinique (MQ), Mexico (MX), Nicaragua (NI), Panama (PA), Peru (PE), Philippines (PH), Puerto Rico (PR), Samoa (WS), Suriname (SR), Thailand (TH), Tonga (TO), United States (US), Venezuela (VE), Singapore (SG), Vietnam (VN).

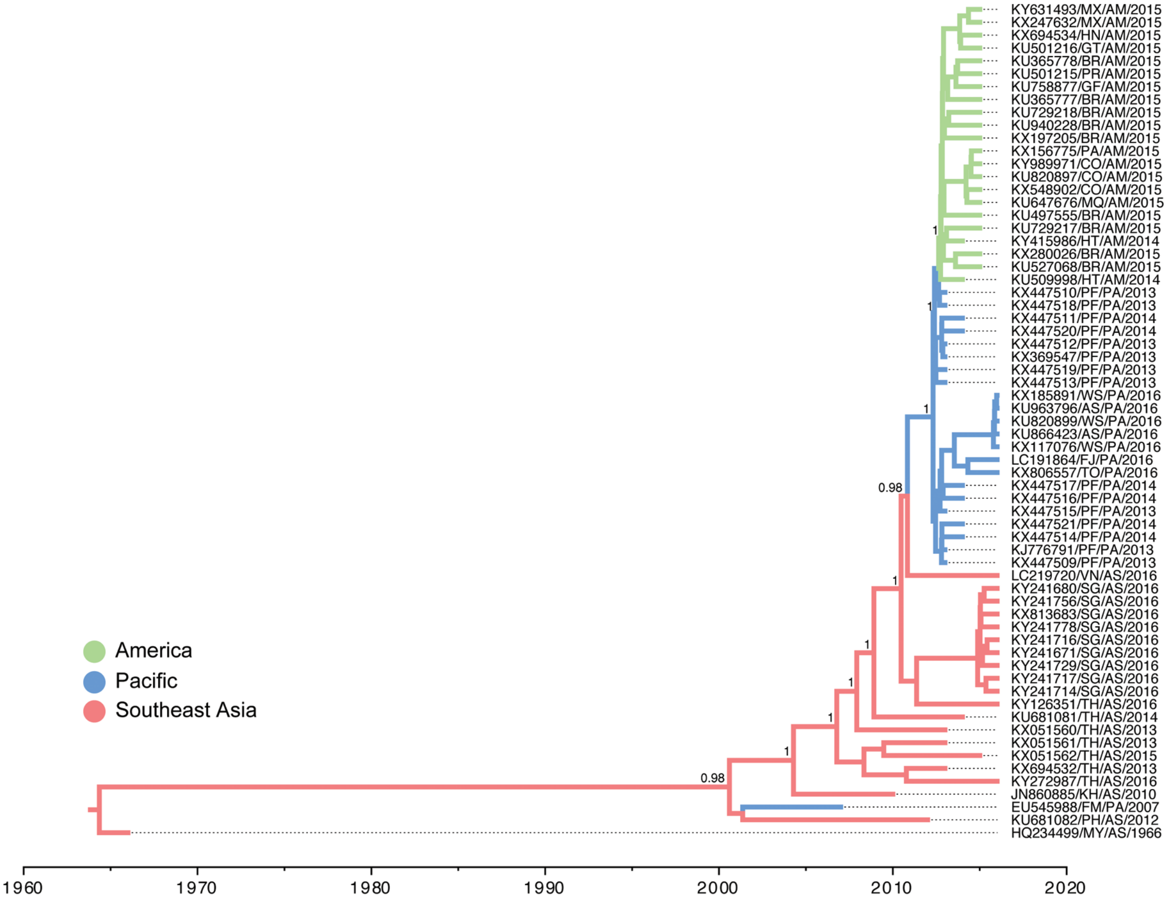


Fig. 2: Bayesian time-scale maximum clade credibility (MCC) phylogenetic tree estimated from Zika virus (ZIKV) Asian genotype genomic sequences using a symmetric phylogeographic model. Branches are coloured according to the most probable location state (geographic region) of their descendent nodes as indicated at the legend on the lower left. Numbers at key selected nodes represent the posterior probability supports of the clades. All horizontal branch lengths are drawn to a scale of years. See legend of Figure for taxon labels information.
